# Neuroprotective effects of lutein in a rat model of retinal detachment

**DOI:** 10.1007/s00417-012-2128-z

**Published:** 2012-08-18

**Authors:** Tiffany T. Y. Woo, Suk-Yee Li, Wico W. K. Lai, David Wong, Amy C. Y. Lo

**Affiliations:** 1 Eye Institute, Li Ka Shing Faculty of Medicine, The University of Hong Kong, Hong Kong, China; 2 Research Centre of Heart, Brain, Hormone & Healthy Aging, LKS Faculty of Medicine, The University of Hong Kong, Hong Kong, China

**Keywords:** Apoptosis, Xanthophyll, Healon, Retinal neuron, Retinal detachment, Lutein, Neuroprotection

## Abstract

**Background:**

Retinal detachment (RD) is a leading cause of blindness, and although final surgical re-attachment rate has greatly improved, visual outcome in many macula-off detachments is disappointing, mainly because of photoreceptor cell death. We previously showed that lutein is anti-apoptotic in rodent models of ischemia/reperfusion injury. The objective of this study is to investigate lutein as a possible pharmacological adjunct to surgery.

**Methods:**

Subretinal injections of 1.4 % sodium hyaluronate were used to induce RD in Sprague–Dawley rats until their retinae were approximately 70 % detached. Daily injections of corn oil (control group) or 0.5 mg/kg lutein in corn oil (treatment group) were given intraperitoneally starting 4 h after RD induction. Animals were euthanized 3 days and 30 days after RD and their retinae were analyzed for photoreceptor apoptosis and cell survival at the outer nuclear layer (ONL) using TUNEL staining and cell counting on retinal sections. Glial fibrillary acidic protein (GFAP) and rhodopsin (RHO) expression were evaluated with immunohistochemistry. Western blotting was done with antibodies against cleaved caspase-3, cleaved caspase-8 and cleaved caspase-9 to delineate lutein’s mechanism of action in the apoptotic cascade. To seek a possible therapeutic time window, the same set of experiments was repeated with treatment commencing 36 h after RD.

**Results:**

When lutein was given 4 h after RD, there were significantly fewer TUNEL-positive cells in ONL 3 days after RD when compared with the vehicle group. Cell counting showed that there were significantly more nuclei in ONL in lutein-treated retinae by day 30. Treatment groups also showed significantly reduced GFAP immunoreactivity and preserved RHO expression. At day 3 after RD, Western blotting showed reduced expression of cleaved caspase-3 and cleaved caspase-8 in the treatment group. No difference was found for cleaved caspase-9. When lutein was given 36 h after RD similar results were observed.

**Conclusions:**

Our results suggest that lutein is a potent neuroprotective agent that can salvage photoreceptors in rats with RD, with a therapeutic window of at least 36 h. The use of lutein in patients with RD may serve as an adjunct to surgery to improve visual outcomes.

## Introduction

Retinal detachment (RD), defined as the separation of the neurosensory retina from the underlying retinal pigment epithelium (RPE), can cause devastating visual loss. With advances in surgical techniques, the anatomical reattachment rate has greatly increased, especially after repeated surgery [1, 2]. However, the final visual outcome remains, in many cases, disappointing once the retinal detachment involved the macula. Even with total retinal reattachment, long-term post-operative VA may lie anywhere in the range of 0.2 to 0.4 [3]. Photoreceptor apoptosis has been postulated to be the main reason for such visual loss [4–6], alongside other structural changes in the retina such as glial scarring [7]. Neuroprotection has thus become a focus of research to achieve better visual outcome by preventing or reducing photoreceptor cell death.

Lutein, a carotenoid macular pigment, has been demonstrated in an NIH sponsored, long-term and large scale randomised placebo-controlled trial to be effective in reducing the risk of developing advanced age-related macular degeneration (AMD). Krinsky has reviewed the biological mechanisms of the protective role of lutein and zeaxanthin in the eye in the context of AMD [8]. Since then, lutein has been studied extensively as an anti-oxidant neuroprotectant and anti-inflammatory agent in different disease models including diabetic retinopathy [9], uveitis [10], light-induced retinopathy [11, 12], and ischemia/reperfusion injury [13].

Lutein has been shown to have effect, not only on RPE, but also on other cellular components including ganglion cells, inner plexiform and nuclear layers, and even photoreceptors. Using TUNEL and cleaved caspase-3 assays, Sasaki et al. have demonstrated an anti-apoptotic effect of lutein in a streptozotocin-induced diabetic mouse model [9]. The effect of lutein in this study appears to be on the retinal ganglion cells and the inner plexiform and nuclear layers, as evidenced by thickness measurements and cell counting. Recently, Sasaki’s group also showed in a light-induced retinopathy mouse model that lutein exerts an anti-apoptotic effect in the photoreceptor layer through inducing DNA repair [12]. Apart from acting on retinal neurons, the effect of lutein has also been demonstrated on neurons in the brain in a rodent stroke model [14].

Lutein has also demonstrated anti-oxidative properties in the eye [8, 13, 15]. It absorbs blue light and contains double bonds that quench reactive oxygen species, therefore reducing oxidative stress. There is some evidence that ROS scavenger and antioxidants may have a protective effect in RD [16–18]. In RD, the separation of the neurosensory retina from the underlying RPE deprived the photoreceptors the supply of oxygen and nutrients, inducing hypoxic stress and in turn generation of reactive oxygen species and oxidative stress [19, 20], eventually causing photoreceptor cell death [21–23]. Lutein protected photoreceptors from oxidative stress-induced cell death [24].

Many AMD patients are currently being prescribed lutein in the form of dietary supplements with tangible improvements in vision [25, 26], and there is a body of literature on its pharmacokinetics. The exact mechanism(s) through which lutein reaches the eye and crosses the blood-retinal barrier has long been an interest of study. Randomized controlled studies evaluating the macular pigment optical density (MPOD) with and without lutein treatment have indirectly confirmed that there is lutein uptake in the retina, as seen from a significant increase of MPOD with lutein administration [27, 28]. But only until recently has new light been shed on how lutein enters the eye. Li et al. have recently identified a lutein-binding protein in the family of steroidogenic acute regulatory domain (StARD) proteins responsible as a ‘receptor’ for lutein to bind to the retina [29]. This lutein-binding protein binds lutein selectively with high affinity and is present in all nuclear layers (outer nuclear layer, inner nuclear layer and ganglion cell layer) of the macular retina. As for how much lutein quantitatively enters the eye remains an issue of debate. Li et al. have, however, established that the retina is the tissue with the highest uptake of lutein, with concentrations up to 1 mM in some human subjects [30].

To date there has been no study of the effect of lutein on rhegmatogenous retinal detachment, a condition that is dominated by cell death in the outer retinal layers. Lutein has a high safety profile when taken in the usual doses over a prolonged period [31]. This, together with its accessibility and bioavailability, makes lutein an attractive medical adjuvant, if it could be shown to have a protective effect on rhegmatogenous retinal detachment, the treatment at present is primarily surgical.

## Methods

### Animals and study design

All experiments were performed in accordance with the ARVO Statement for the Use of Animals in Ophthalmic and Vision Research and approved by the Committee on the Use of Live Animals in Teaching and Research of the University of Hong Kong (CULATR 1941-09). Male Sprague–Dawley (SD) rats (200–250 g) were kept in a temperature-controlled room with a 12-hour light/12-hour dark cycle. Rats were divided into two groups—namely, a treatment group receiving lutein in corn oil and a vehicle group receiving corn oil alone. Retinal detachment was induced in right eyes (OD), while left eyes (OS) served as control. Four separate experiments were carried out (Table 1): the first two evaluated the short-term and long-term effects of administering lutein 4 h after retinal detachment while the second two evaluated the short-term and long-term effects of commencing lutein treatment 36 h after RD.

**Table 1:** Design of experiments

Experiment	Commencement of treatment (hours after RD)	Treatment	Euthanasia (days)
1	4	Daily	3
2	4	Daily for first 3 days then once every 3 days	30
3	36	Daily	3
4	36	Daily for first 3 days then once every 3 days	30

### Experimental model of retinal detachment

Rats were anesthetized with intraperitoneal injections of a mixture of xylazine (7 mg/kg) and ketamine (70 mg/kg). Topical anesthetics (Alcaine; 1 % proparacaine hydrochloride, Alcon) and mydriatics (Mydriacyl; 1 % tropicamide, Alcon) were applied to the right eye. A small keratotomy was created to allow release of intra-ocular pressure (IOP) during the procedure. The fundus was visualized under a surgical microscope (M620, Leica, Switzerland) with Gel 4000 (Bruschettini, Italy) applied on the eye and a round cover slip as a surrogate contact lens.

**Fig. 1:**
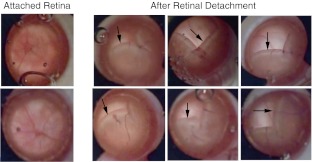
Representative images showing fundi of un-operated eyes (*left*) and eyes with experimental retinal detachment (*right*). Note that all retinae were at least 70 % detached

Retinal detachment was induced mechanically in the right eyes of rats through a superonasal sclerotomy and retinotomy approximately 2 mm below the limbus using a 30-gauge needle. With the retinotomy simulating a retinal tear, the needle was then retracted to the subretinal space where Healon (1.0 % sodium hyaluronate, AMO) was injected until 70 % of the retina was detached, mimicking a rhegmatogenous retinal detachment. Detachment was confirmed visually under the surgical microscope and a fundus photograph was taken for each animal (Fig. 1). Prophylactic topical antibiotics (Tobrex®; tobramycin ointment, Alcon) were applied to prevent post-operative infection. Animals were monitored periodically until they regained consciousness, and daily post-operatively.

### Treatment administration

Lutein 20 % fluid suspension (FS), containing 20 % microionized crystals of lutein extracted from *Tagetes erecta* dispersed in corn oil, was obtained from DSM Nutritional Products (Netherlands). Treatment groups were given 0.5 mg/kg lutein diluted to 0.3 mg/ml with pure corn oil (C8267; Sigma Aldrich Co., St Louis, MO). Vehicle groups were given comparable amounts of corn oil. All treatments were administered intraperitoneally.

### Tissue collection and processing

Animals were euthanized with pentobarbitone overdose at various time points. Eyes were examined for height of RD and a 6-0 vicryl suture (Johnson & Johnson, USA) was attached to the nasal conjunctiva of each eye to mark the anatomical position. Eyes were enucleated and the cornea and lens were carefully removed. Eyeballs were fixed overnight at 4 °C with 4 % paraformaldehyde in phosphate buffered saline (PBS, 0.01 M, pH 7.4). They were then dehydrated with a graded series of ethanol and chloroform, embedded with suture (nasal part) upwards in paraffin wax and cut into 6 μm sagittal sections using a microtome (Microm HM 315R, Heidelberg, Germany).

### Histological analysis

Retinal sections were deparaffinized and stained with hematoxylin & eosin (H&E). Outer nuclear layer (ONL) thickness and cell count per unit area were quantified in three separate positions: central (100–150 μm from optic nerve), peripheral (100–150 μm from ora serrata) and mid-peripheral (half-way in between central and peripheral). Two representative sections were chosen randomly for each eye, from which measurements were taken and their values averaged. Only retinal sections showing optic discs were included for analysis. Areas that were not detached were excluded.

### TdT-medicated dUTP Nick-End Labeling (TUNEL) assay

TUNEL assay (DeadEnd Fluorometric TUNEL system, Promega, Madison, WI) was performed according to manufacturer’s instructions for quantification of apoptotic cells. Two representative sections were chosen randomly for each eye. Similarly, only retinal sections showing optic discs were selected. Fourteen areas were photographed on each retinal section under 400× magnification, covering almost the entire length of the retina. Sections were counterstained with 4′, 6-diamidino-2-phenylindole (DAPI), a nuclear staining. Cells in the ONL co-stained by both TUNEL and DAPI were counted as apoptotic cells. An apoptotic ratio (number of TUNEL positive cells ÷ total ONL area) was obtained for each eye. Since it has been shown that maximum apoptosis occurs at 3 days after retinal detachment [4], TUNEL staining was performed only for experiments 1 and 3.

### Immunohistochemistry

Three retinal sections showing the optic disc were selected randomly for each eye. Deparaffinized retinal sections were immersed in proteinase K solution for antigen retrieval and blocked in 10 % goat serum for one hour. They were then incubated with the following primary antibodies at 4 °C overnight: rabbit anti-glial fibrillary acidic protein (GFAP, 1:500; Z0334 Dako, Carpinteria, CA), mouse anti-glutamine synthetase (GS, 1:1,000; MAB302 Millipore, Billerica, MA) and rabbit anti-rhodopsin (RHO, 1:20,000; O4886 Sigma-Aldrich Co, St. Louis, MO). Upon thorough washing with PBS, sections were put in their corresponding secondary antibodies (1:500; Molecular Probes, Invitrogen Corporation, Carlsbad, CA) for 1 h at room temperature, after which they were counterstained with DAPI, washed again, cover-slipped, and examined under a fluorescent microscope (Eclipse 80i; Nikon, Tokyo, Japan). Retinal sections were graded from 1 to 5 for intensity of GFAP and rhodopsin immunoreactivity, with 5 being the strongest. Graders were blinded from the treatment grouping to eliminate observer bias.

### Western blot analysis

To delineate the mechanistic pathway by which lutein exerts its anti-apoptotic action, Western blotting was performed for several key players along the apoptotic cascade. We first looked at the level of cleaved caspase-3 activity, an important effector caspase in the final common pathway in apoptosis. We then looked further upstream at cleaved caspase-8 and cleaved caspase-9 activity for lutein involvement in the Fas-mediated extrinsic pathway and mitochondrial intrinsic pathway respectively.

Experiments 1 and 3 were repeated to obtain protein samples for Western blotting. Experimental and control retinae from both treatment and vehicle groups were separated from vitreous and RPE, and subsequently homogenized in 150 ml RIPA lysis buffer. In experimental eyes, attached parts of the retinae were manually dissected and excluded from analysis. Samples were centrifuged at 13,500 rpm in 4 °C for 25 min, and the supernatant were aspirated and placed into fresh tubes. The protein concentration of each sample was determined with a spectrophotometer, and absorbance was plotted against a protein standard. Protein samples were then separated with 15 % SDS-PAGE gels and transferred to PVDF membranes. Blocking was done with 5 % non-fat milk in TBST. Membranes were incubated with primary antibodies against cleaved caspase-3 (1:1,000; #9661; Cell Signaling Technology Beverly, MA), cleaved caspase-8 (1:2,000; #9429; Cell Signaling Technology), cleaved caspase-9 (1:2,000; #9507; Cell Signaling Technology), Bcl-2 (1:2,000; #2876; Cell Signaling Technology) and phospho-Akt (1:1,000; #4051; Cell Signaling Technology) at 4 °C overnight. For internal control, membranes were also incubated with antibody against actin (1:10,000; MAB1501; Chemicon, Temecula, CA) for 30 min in room temperature. Protein bands were detected with Amersham ECL Detection Reagents (Amersham Pharmacia Biotech, Arlington Heights, IL), except those for cleaved caspase-3, which were detected with Amersham ECL Plus Detection Reagents (Amersham Pharmacia Biotech, Arlington Heights, IL). Densitometry was performed with Image J (1.43u, National Institutes of Health, USA), and protein levels were normalized against actin levels.

### Statistics

Data were presented as mean ± standard error of mean (SEM). Statistical analysis for ONL thickness, cell counting and TUNEL was performed with SPSS using independent samples *t*-test. Statistical analysis for immunohistochemistry was performed with SPSS using Mann Whitney *U* test. Statistical analysis for Western blot results was performed with SPSS software (IBM corporation, USA) using one-way ANOVA with post-hoc Tukey’s test.

## Results

### Retinal detachment model

All detached retinae remained detached until the day of euthanasia. The percentage of detachment (∼70 %) was documented by fundus photographs (Fig. 1). The number of animals used in each experiment is shown in Table 2. Post-operative complications detected include minor bleeds associated with the injection and transient increases in IOP.

**Table 2:** The number of animals used in each experiment

Experiment	Immunohistochemistry			Western blotting		
	Vehicle	Lutein	Total	Vehicle	Lutein	Total
1	*n* = 10	*n* = 11	21	*n* = 11	*n* = 11	22
2	*n* = 12	*n* = 10	22			
3	*n* = 9	*n* = 9	18	*n* = 11	*n* = 11	22
4	*n* = 8	*n* = 10	18			

### ONL apoptosis

**Fig. 2:**
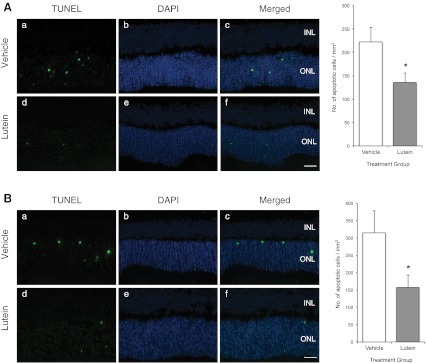
**A** Experiment 1; **B** Experiment 3. Representative photomicrographs of retinal sections after TUNEL staining (*a*, *d*) and counter-staining with DAPI (*b*, *e*). *Green dots* represented TUNEL-positive cells, and *blue staining* represented DAPI-positive nuclei in the ONL. Merged images (*c*, *f*) showed co-localization of apoptotic cells with nuclei in the ONL. No apoptotic cells were found in other layers. Quantification showed approximately 50 % decrease in apoptotic cells. (*p < 0.05). *Scale bar*, 25 μm

TUNEL-positive nuclei were found to be present in the ONL of the detached retina and confirmed by co-localization of photoreceptor nuclei using DAPI staining. In experiment 1, treatment with lutein reduced the number of apoptotic cells/mm^2^ significantly when compared with that of the vehicle-treated group (*p* < 0.05; Fig. 2A). Similar results were obtained in experiment 3 when treatment was withheld until 36 h after onset of RD (*p* < 0.05; Fig. 2B).

### ONL cell counting

Cell counting of the ONL showed no significant difference between lutein- and vehicle-treated groups for experiments 1 and 3 (data not shown).

**Fig. 3:**
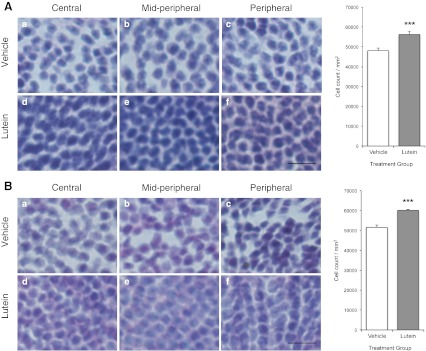
**A** Experiment 2; **B** Experiment 4. Representative photomicrographs of H&E-stained retinal sections showing vehicle- (*a*, *b*, *c*) and lutein-treated (*d*, *e*, *f*) ONL. Vehicle-treated retinae appeared to have more widely spaced nuclei, with loss of the compact architecture normally observed. The lutein-treated retinae appeared to have more closely packed nuclei resembling non-detached retains. Similar results were seen in central (*a*, *d*), mid-peripheral (*b*, *e*) and peripheral (*c*, *f*) positions of the retinae. Quantitative analysis showed significantly greater number of cells in lutein-treated groups compared with vehicle-treated groups. (***p = 0.001). *Scale bar*, 10 μm

However, in experiment 2, a significantly greater number of cells were found remaining in the ONL of lutein-treated animals than vehicle-treated ones (*p* = 0.001; Fig. 3A). Cell counting showed that there were approximately 65,304 ± 1,843 cells per mm^2^ in contralateral undetached retina (of all treated rodents), 58,202 ± 1,054 cells per mm^2^ in the detached retina of lutein treated eyes and 49,843 ± 1,195 cells per mm^2^ in vehicle-treated detached retina. Similar results were found in experiment 4 when treatment was given 36 h after RD, with lutein-treated animals also showed significantly more surviving cells in the ONL (*p* < 0.001; Fig. 3B).

### Rhodopsin

**Fig. 4:**
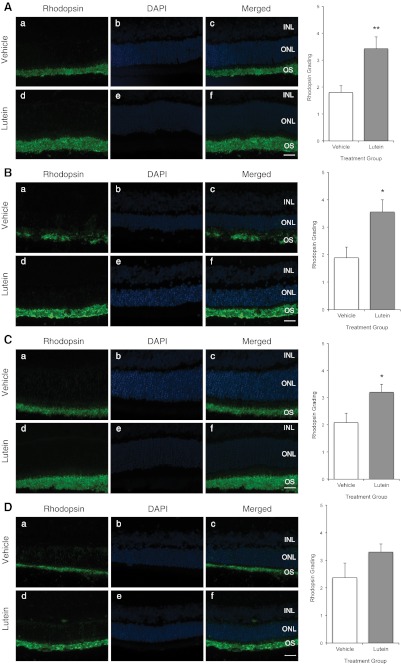
**a** Experiment 1; **b** Experiment 2; **c** Experiment 3; **d** Experiment 4. Representative photomicrographs of retinal sections after rhodopsin staining (*a*, *d*) and counter-staining with DAPI (*b*, *e*). *Green staining* represented rhodopsin immunopositivity, and *blue staining* represented DAPI-positive nuclei in the ONL. Rhodopsin staining was stronger both in intensity and thickness in lutein-treated retinae compared with vehicle-treated retinae. Merged images (*c*, *f*) showed rhodopsin immunoreactivity localized to the outer segment. Semi-quantification using subjective grading out of 1 to 5 showed significant preservation in rhodopsin grading in lutein-treated retinae. (**p = 0.01). (*p < 0.05). *Scale bar*, 25 μm

In experiments 1 and 3, treatment with lutein resulted in relatively preserved rhodopsin (RHO) immunoreactivity both in terms of thickness and intensity when compared with vehicle-treated groups whether given 4 h (*p* = 0.01; Fig. 4A) or 36 h after RD (*p* < 0.05; Fig. 4C). Likewise, results from experiments 2 (p < 0.05; Fig. 4B) and 4 (*p* > 0.05; Fig. 4D) showed a similar trend, but was however not statistically significant in experiment 4 possibly due to large variance.

### GFAP

**Fig. 5:**
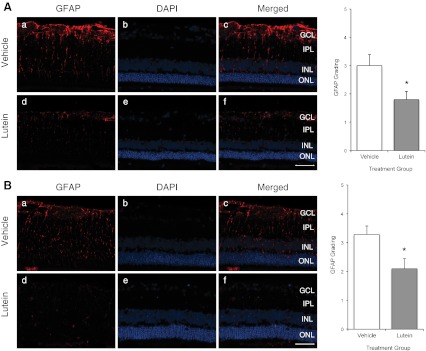
**A** Experiment 2; **B** Experiment 4. Representative photomicrographs of retinal sections after GFAP staining (*a*, *d*) and counter-staining with DAPI (*b*, *e*). *Red staining* represented GFAP immunopositivity, and *blue staining* represented DAPI-positive nuclei in the ONL. GFAP staining appeared to show thick Muller cell processes that span across the entire thickness of the retina in vehicle-treated (*a*, *b*, *c*) retinae, while only limited GFAP-immunoreactivity was seen in lutein-treated (*d*, *e*, *f*) retinae. Merged images (*c*, *f*) showed GFAP immunoreactivity mainly at the GCL with extension to the outer layers. Semi-quantification using subjective grading out of 1 to 5 showed significantly decreased GFAP grading in lutein-treated retinae. (*p < 0.05). *Scale bar*, 50 μm

GFAP immunoreactivity was mainly present in the ganglion cell layer (GCL) with varying degrees of extension into the outer retina, indicating proliferating Muller cell processes. Our results showed that in the first 3 days of RD, treatment with lutein or corn oil produced no tangible difference in glial activation (data not shown). However, in experiments 2 and 4, lutein treatment significantly reduced GFAP-immunoreactivity, both when given 4 h and 36 h after RD. (*p* < 0.05; Fig. 5A and B). Notably, both length and thickness of Muller cell processes were found to be significantly greater in the vehicle-treated group when compared with the lutein-treated group, indicating astrocytic hypertrophy and proliferation.

### Apoptotic pathway

**Fig. 6:**
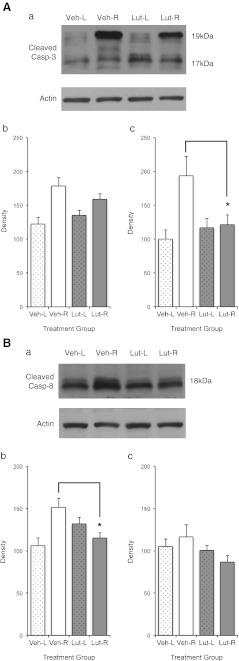
**a** Cleaved caspase-3; **b** Cleaved caspase-8. Western blot analysis (*a*) with normalized densitometry quantifications of activity using eyes from experiment 1 (*b*) and experiment 3 (*c*). *Veh-L* vehicle left control eyes; *Veh-R* vehicle right RD eyes; *Lut-L* lutein left control eyes; *Lut-R* lutein right RD eyes. Significant reduction of cleaved caspase-3 level was seen in experiment 3, but the reduction was not statistically significant in experiment 1. Significant reduction of cleaved caspase-8 level was seen in experiment 1, but the reduction was not statistically significant in experiment 3. (*p < 0.05)

The group treated 36 h after RD showed significantly lower cleaved caspase-3 level than the vehicle-treated group (*p* < 0.05; Fig. 6A-c). The group treated 4 h after RD showed similar results, although the trend of decrease was not statistically significant (*p* > 0.05; Fig. 6A-b). Significant difference in cleaved caspase-8 level was also found between animals treated with lutein and vehicle starting 4 h after RD (*p* < 0.05; Fig. 6B-b). Again, a similar trend was observed for the group receiving treatment 36 h after RD, but the difference was not of statistical significance (*p* > 0.05; Fig. 6B-c). The level of cleaved caspase-9 was also determined, but no statistically significant difference was found between the two groups (data not shown).

## Discussion

Previous research showed that cell death starts as early as 12 h [5] after disruption of retina-RPE homeostasis, peaks at day 3 and eventually declines to a basal level [4, 32]. From initial presentation, to a general practitioner, and subsequent referral to an ophthalmologist to the eventual surgical treatment, the duration usually spans more than 1 day. Crucial time would be lost after diagnosis and before surgery that could influence the final visual outcome.

In our study, we designed the first set of experiments (1 and 2) to evaluate the efficacy of lutein as an acute treatment by administering it 4 h after RD. We know clinically that patients rarely present within hours of the onset of symptoms [33]. In view of this, we designed a second set of experiments (3 and 4) to seek a therapeutic window for treatment administration.

### Anti-apoptotic effect

Apoptosis is modulated by a wide range of cell signals, of which the caspases constitute a central role. The two major signaling cascades—the Fas-mediated extrinsic and mitochondrial intrinsic pathways [34]—eventually converge to form a final common effector pathway involving caspases 3, 6 and 7 [35]. It has become increasingly clear in recent years that both pathways are involved in photoreceptor cell death after RD [36]. Zacks et al. suggest that RD induces apoptosis in two phases: the initial phase (both Fas-dependent and -independent), as documented by TUNEL assays, utilizes preexisting stores of proapoptotic proteins; and a second Fas-dependent phase relying on transcription of Fas-pathway intermediates [37]. In our TUNEL studies, the rate of cell death at day 3 (5 %) is in agreement with previous studies [38]. This percentage was relatively small. In other words, 95 % of the photoreceptors would still be intact. Therefore, at day 3, we did not expect to see a significant difference in cell number between the lutein- and vehicle-treated retinae. Further apoptosis is said to peak at day 3. Therefore, in order to find the cumulative effect of any possible anti-apoptotic therapy, instead of counting TUNEL positive cells, one would need to count the total number of surviving cells or nuclei in the ONL. Indeed, we found in our study that by day 30, however, the number of cells that survived changed considerably. In the vehicle-treated group, the average number of viable cells in the ONL was 49,843 cells/mm^2^. Compared to the mean number in the fellow eyes of all treated (lutein or vehicle) rodents of 65,304 cells/mm^2^, roughly 18,023 cells/mm^2^ (23.7 %) were lost, representing nearly a quarter of the usual cell density. In contrast, we found the lutein-treated retina had a mean cell count of 58,202 cells/mm^2^. The cell loss was reduced significantly from a 23.7 % to around 10.9 % (Fig. 3a). It could be said that lutein prevented around 54 % of cell death.

Our Western blotting results showed that there was a reduction of cleaved caspase-8, indicating that lutein probably acted on the Fas-mediated extrinsic pathway. Previous studies have shown that cleaved caspase-9 activity peaks at 24 h post-injury [36]. The fact that no reduction in cleaved caspase-9 was observed could indicate that lutein had no effect on the intrinsic pathway, but equally it could simply indicate that our sampling time of 72 h was not optimal to detect any change. More research is warranted to elucidate the point of intervention of lutein along the apoptotic cascade.

In fact, apoptosis is only one of the mechanisms through which photoreceptors die in the disease process of RD—others being necroptosis, autophagy and inflammatory processes [6]. The data presented in this study cannot rule out the possibility of lutein involvement in these mechanisms of photoreceptor cell death after RD.

### Muller cell activation and proliferation

GFAP, a marker for reactive gliosis, reflects Muller cell activation caused by RD-induced ischemia [38]. In excess, it can lead to obvious hazardous repercussions such as epiretinal membrane formation, proliferative vitreoretinopathy (PVR) and subretinal glial scarring. This further impairs visual prognosis by reducing the number and integrity of remaining photoreceptors, and inhibiting regeneration of outer segments [39]. A reduction in GFAP-immunoreactivity 30 days post-RD as shown suggested a reduction in glial activation when lutein was given both at 4 or 36 h after RD. This reduction was not observed at 3 days.

### Outer segment function

Rhodopsin, a naturally occurring pigment in the eye, plays a pivotal role in light perception and acts as a marker for retinal function. It is a sensitive indicator for the length and functionality of the outer segment (OS), and carries implications to OS recovery [40, 41], since rhodopsin molecules appear to redistribute after RD in preparation for future use after reattachment [42]. Our results showing higher RHO immunoreactivity in lutein-treated groups provide encouraging data to suggest the possibility that lutein produces a better functional outcome. However, more functional studies with quantitative electroretinography (ERG) must be done to convincingly prove this point.

### Lutein

We administered lutein intraperitoneally to ensure accurate dosing in animals and for convenience in administration. It has already been shown that lutein when taken orally does have a high bioavailability in humans and can achieve concentrations comparable to that of intraperitoneal injections [43, 44].

## Conclusion

We are among the first to show that a safe, convenient, systemic therapy exerts anti-apoptotic and neuroprotective effects on retinal ganglion cells in an ischemic/reperfusion model. In this study, we used an established Healon-induced retinal detachment model and showed that photoreceptors could also be salvaged with lutein given systematically employing the same doses per kg as that used for human daily dietary supplements.
